# Supplementary material for: Equine Endothelial Cells Show Pro-Angiogenic Behaviours in Response to Fibroblast Growth Factor 2 but Not Vascular Endothelial Growth Factor A
Source: Int J Mol Sci. 2024 May 30;25(11):6017. doi: 10.3390/ijms25116017 (PMC11172845; doi:10.3390/ijms25116017)

Figure S3d. pERK

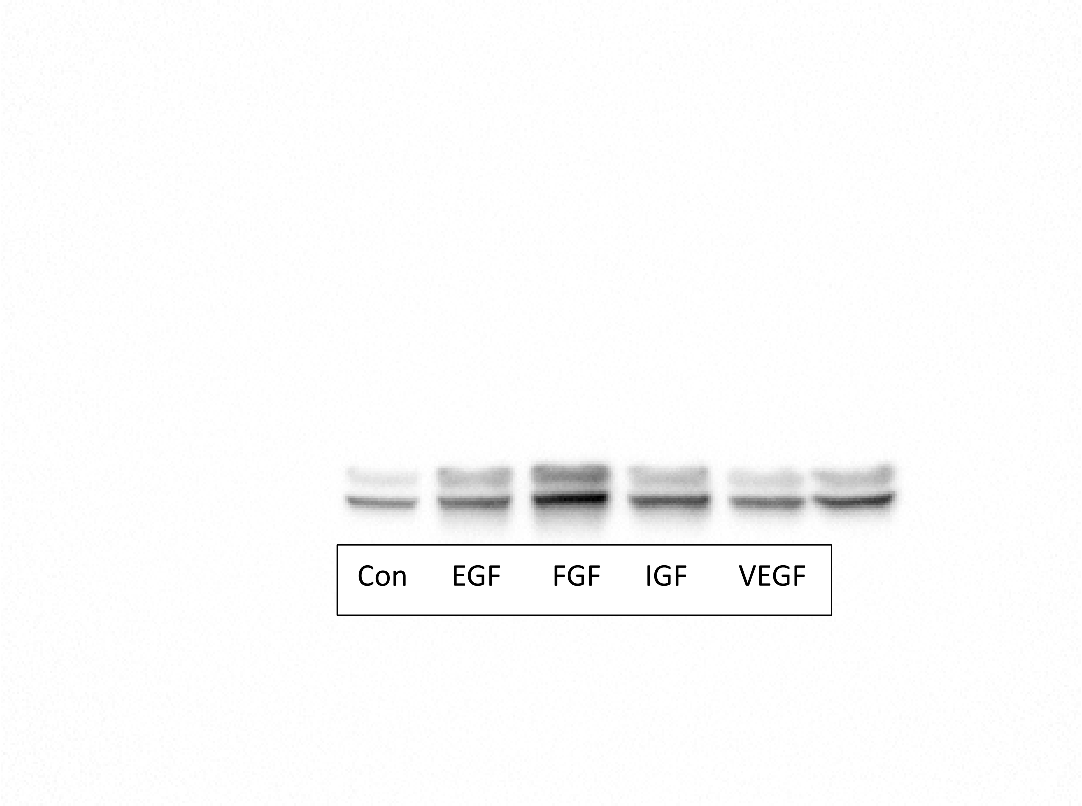

Figure S3d. Total ERK

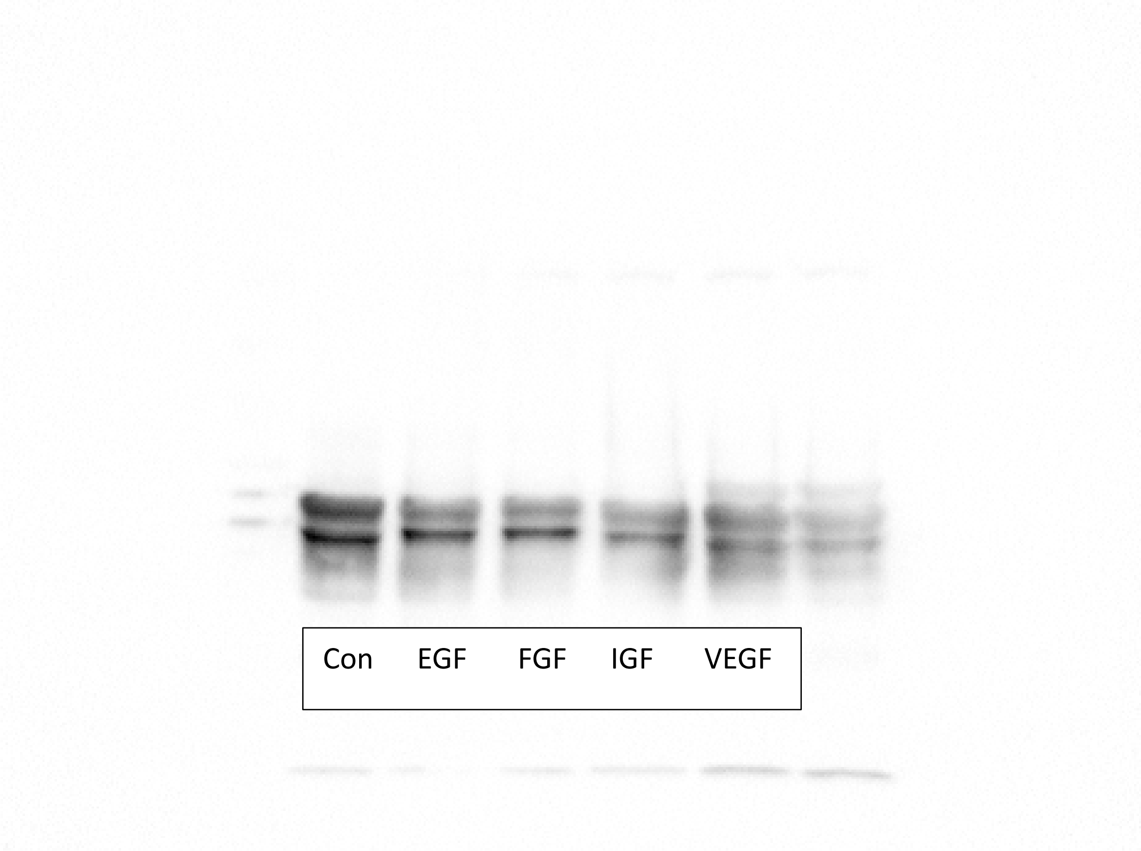

Figure S4d. pERK

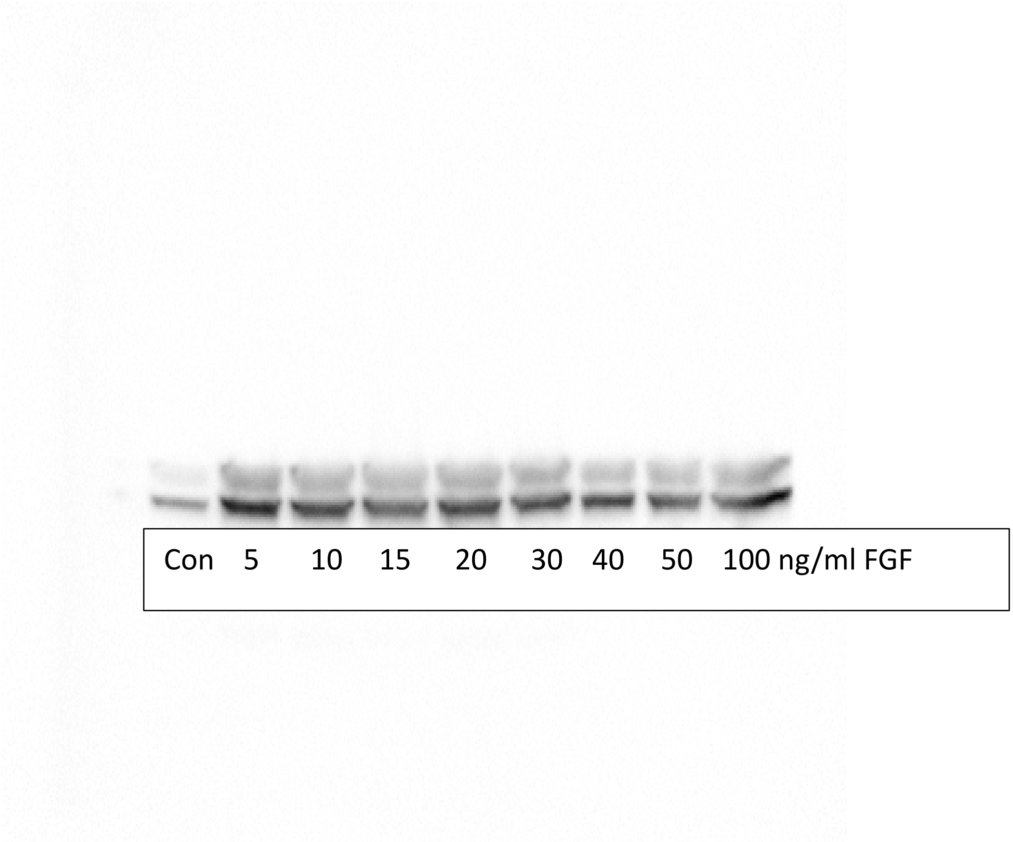

Figure S4d. Total ERK

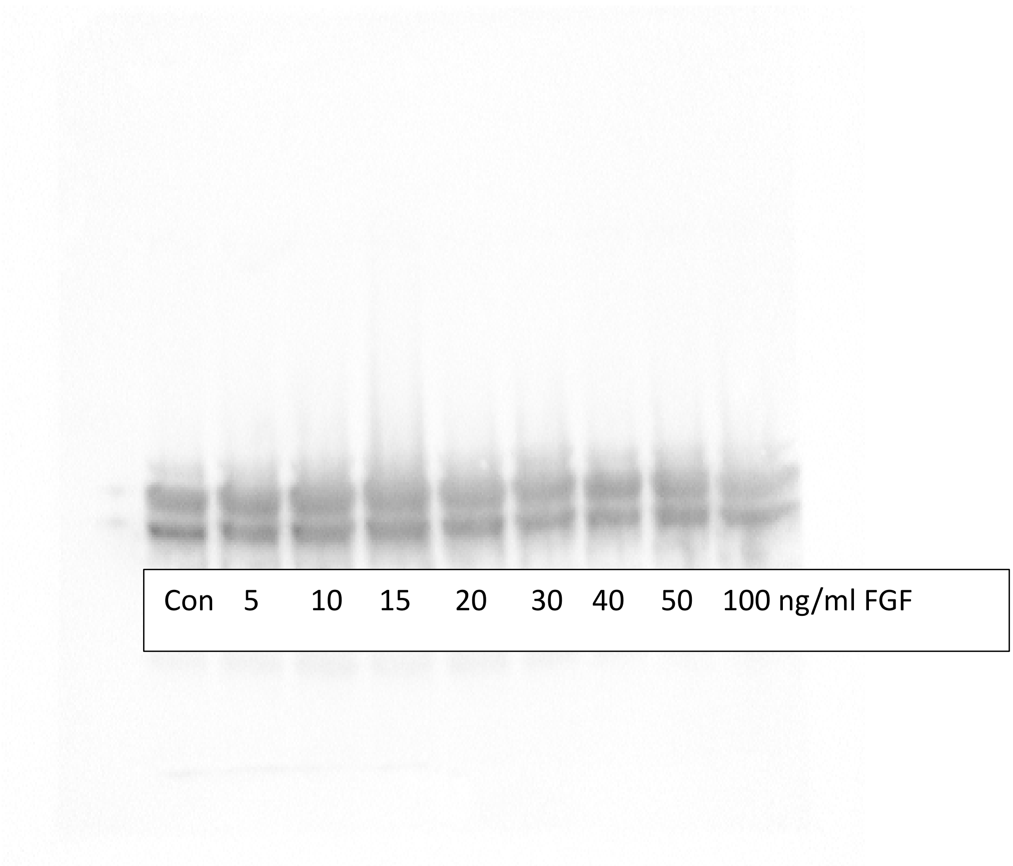

Figure S5d pERK

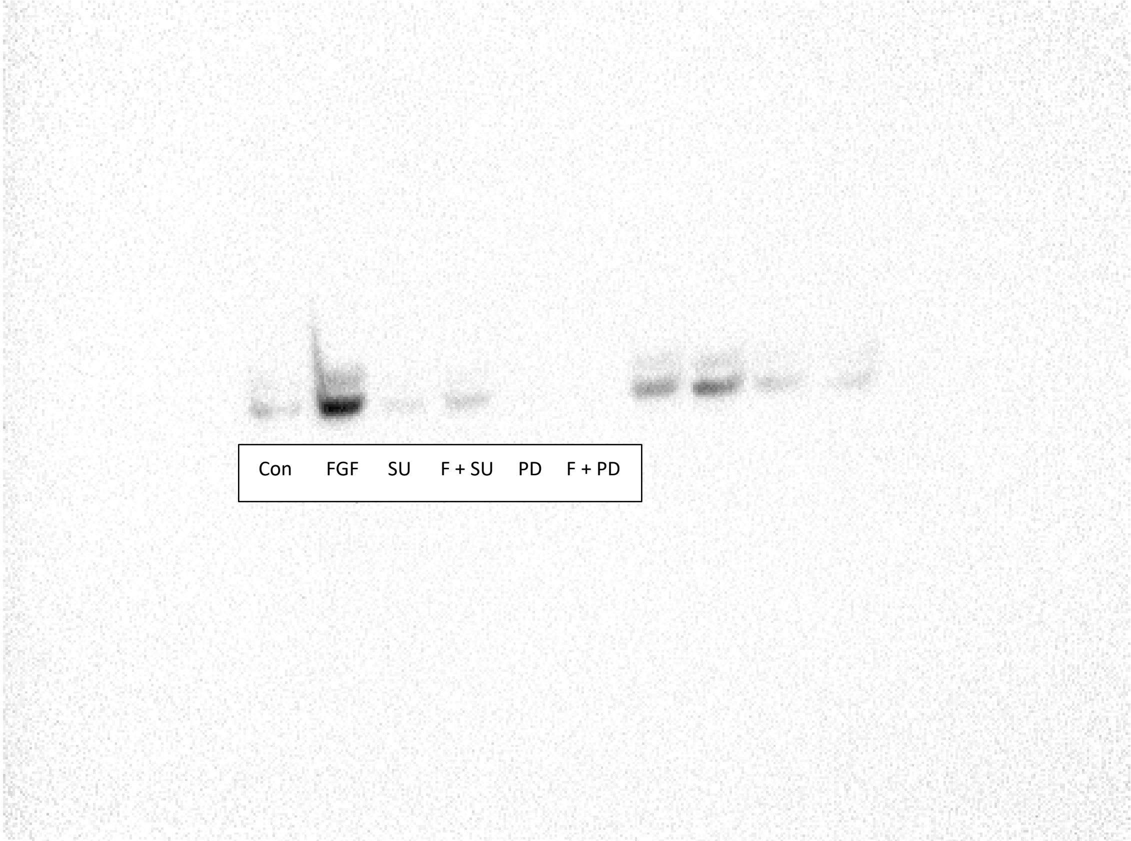

Figure S5d Total ERK

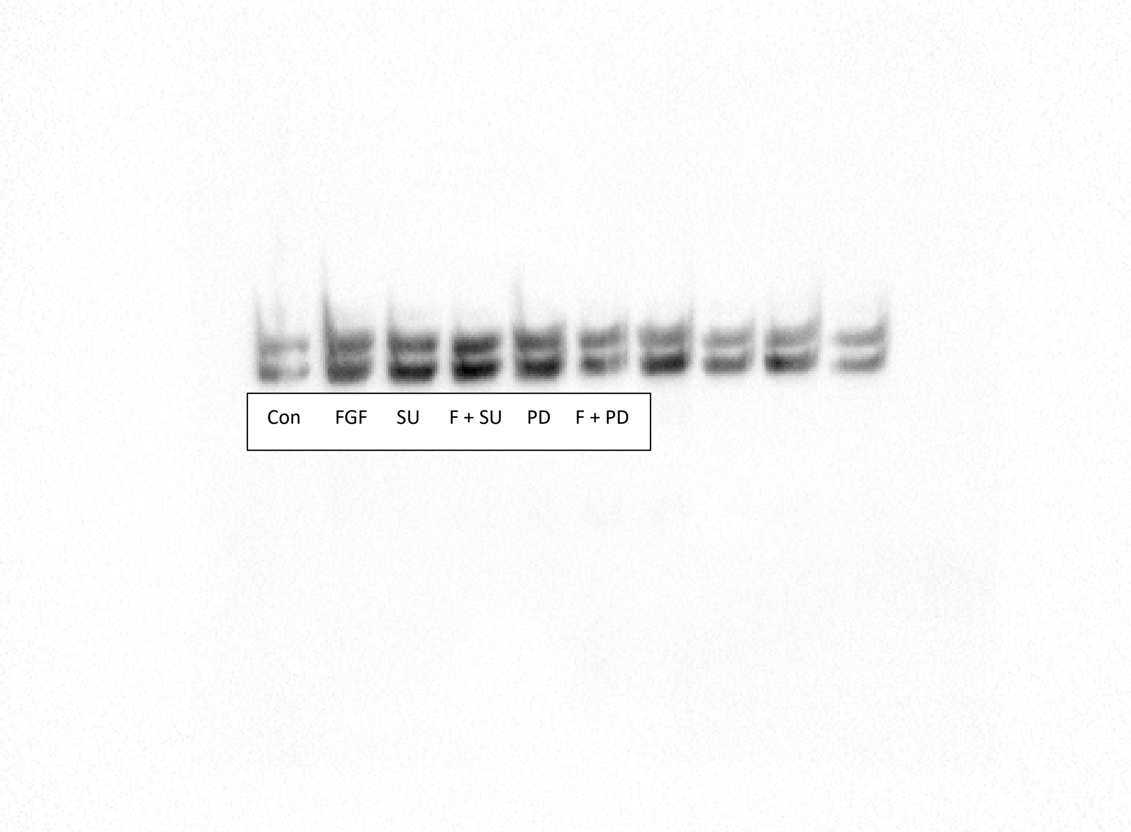

Figure S5d B actin

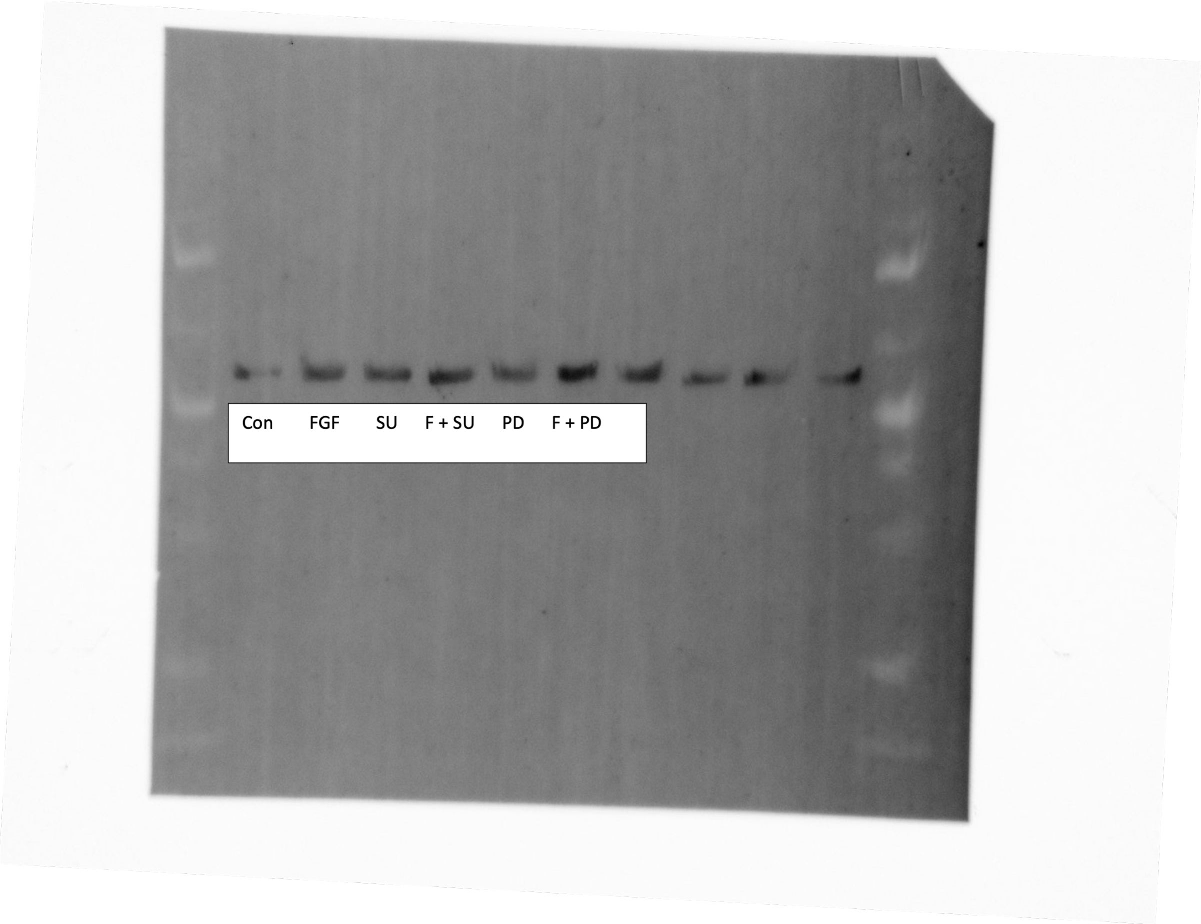
Con
FGF
SU
F + SU
PD
F + PD

Figure S6d pERK

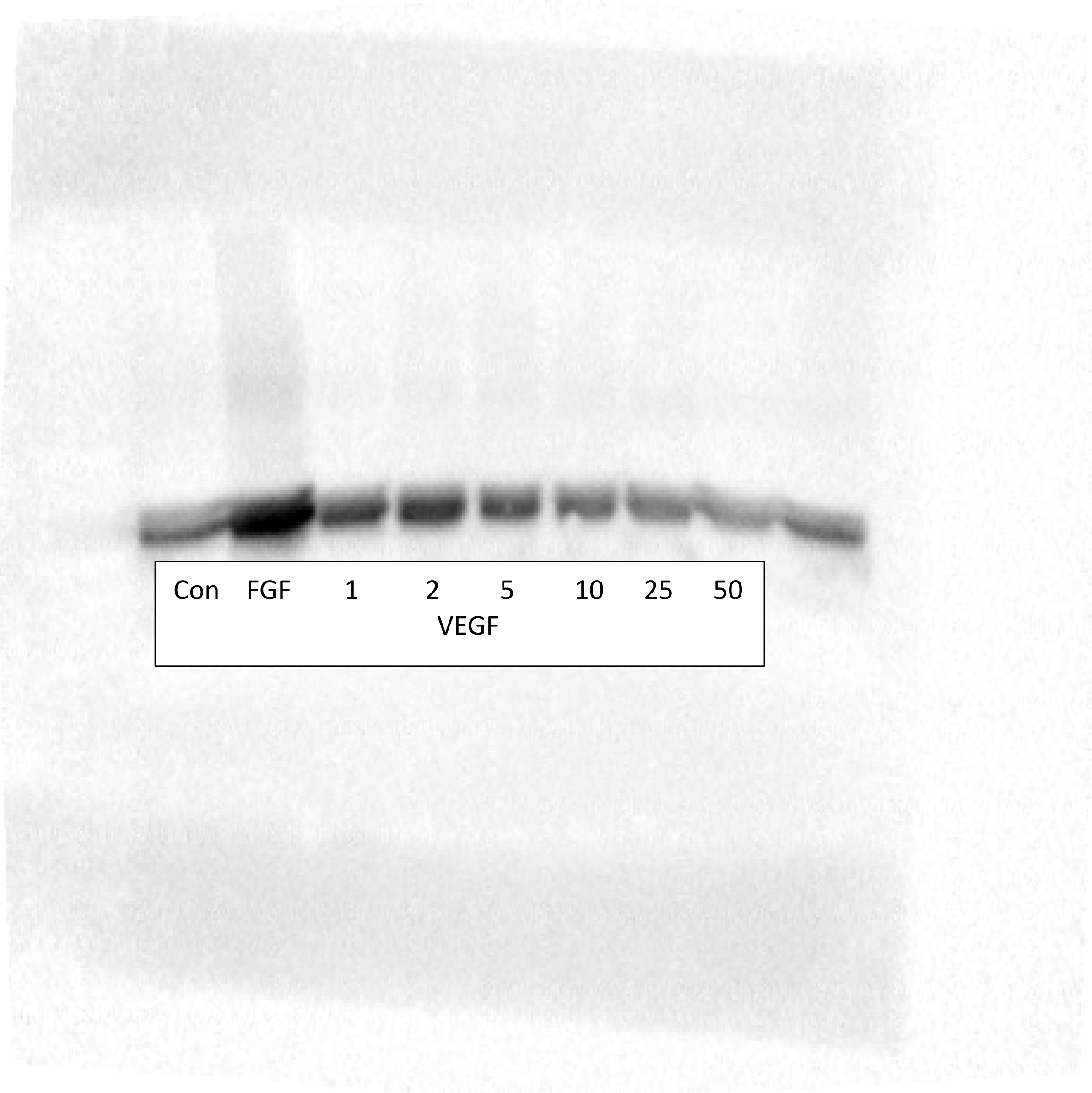

Figure S6d B actin

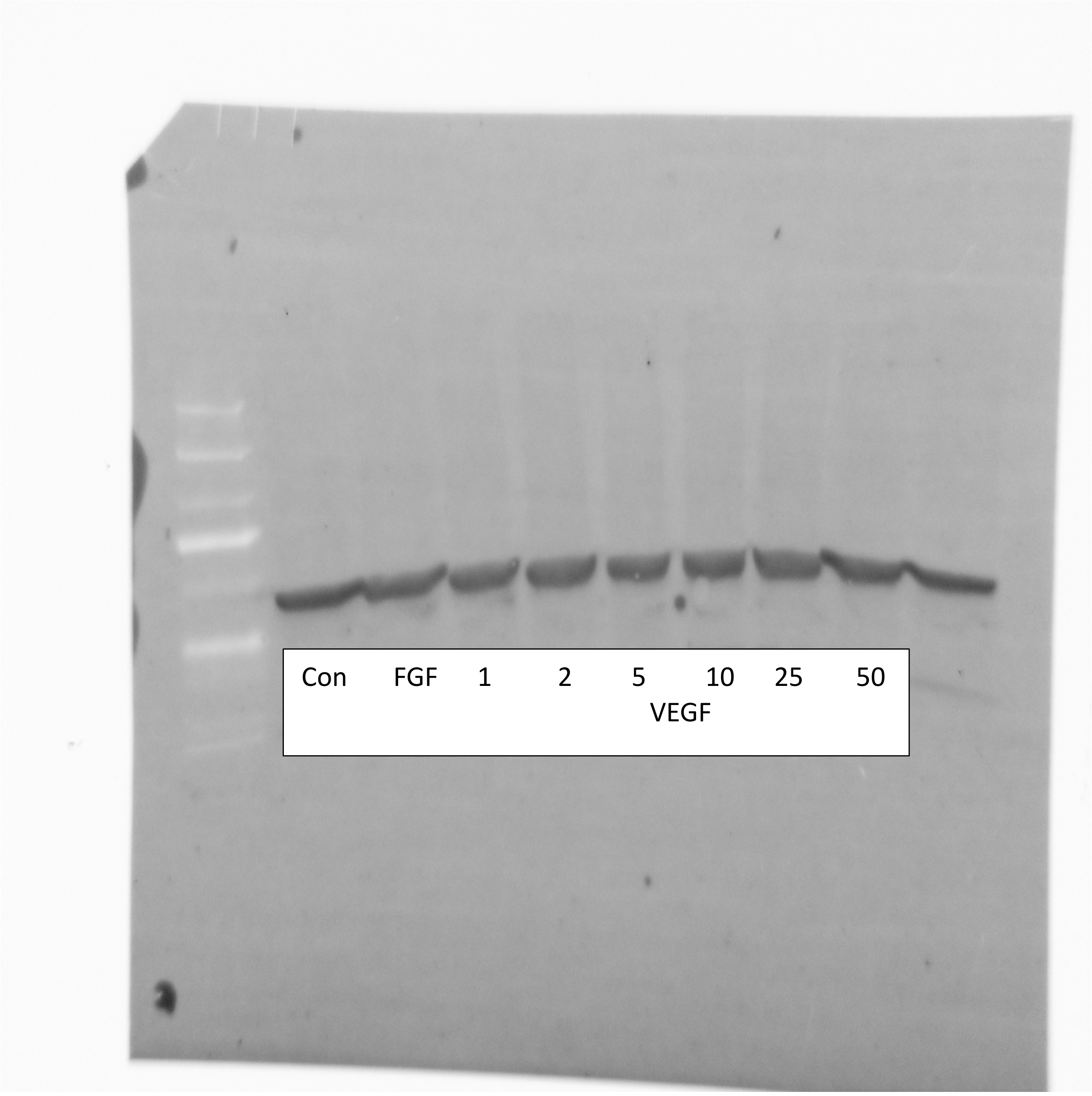

Supplemental figure S6 pERK

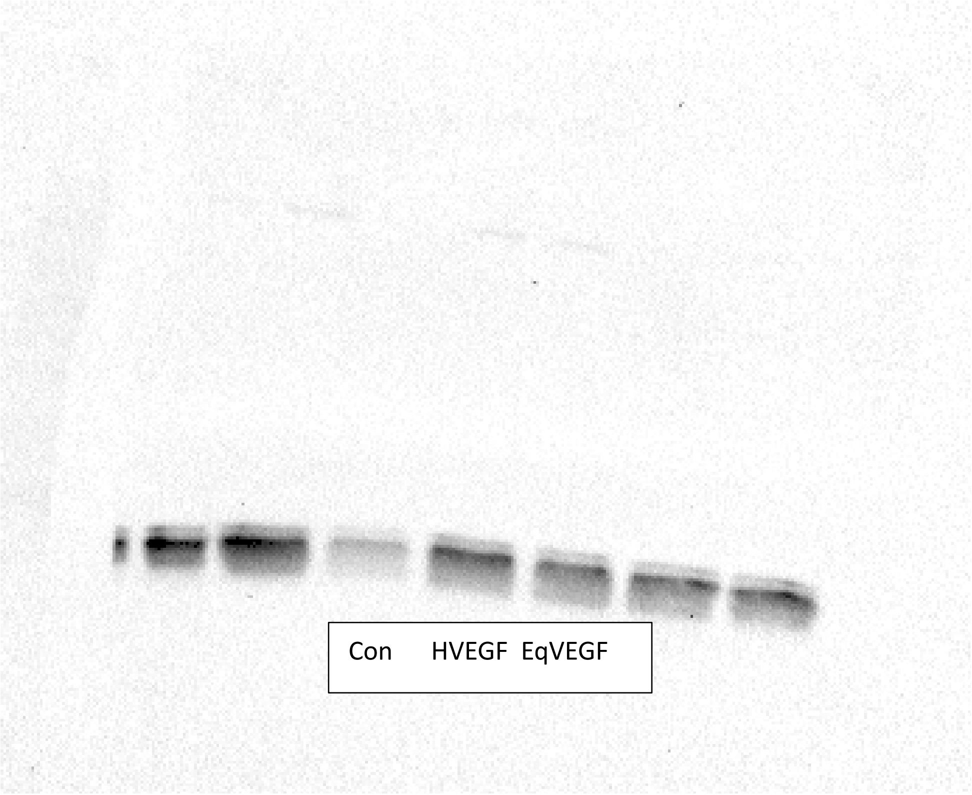

Supplemental figure S6 Total ERK

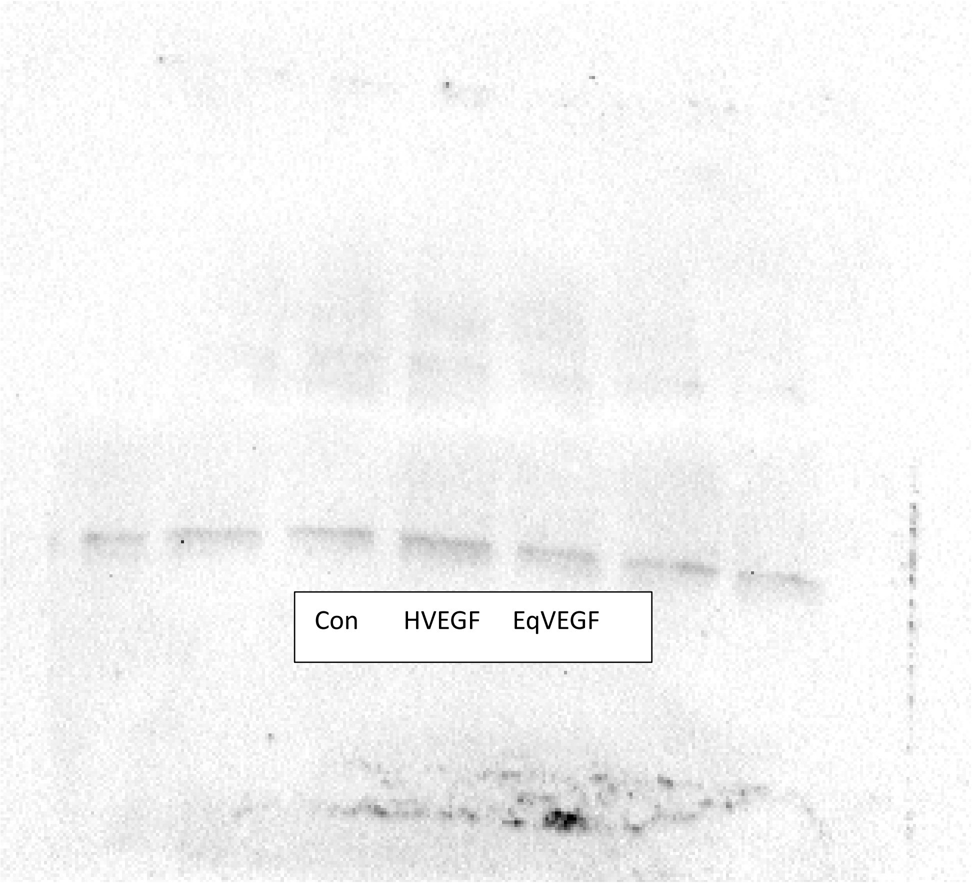

Supplement: Supplementary file 1 [file ijms-25-06017-s001.zip › ijms-2938826-supplementary/Original blots.pdf]
