# Supplementary material for: Equine Endothelial Cells Show Pro-Angiogenic Behaviours in Response to Fibroblast Growth Factor 2 but Not Vascular Endothelial Growth Factor A
Source: Int J Mol Sci. 2024 May 30;25(11):6017. doi: 10.3390/ijms25116017 (PMC11172845; doi:10.3390/ijms25116017)
Supplement: Supplementary file 1 [file ijms-25-06017-s001.zip › ijms-2938826-supplementary/Supplemental materials IJMS.pdf]

Supplementary Methods

Cell isolation by collagenase digestion

Following initial aortic dissection and longitudinal incision, collagenase solution (0.25 mg/ml in Medium 199 with Earle's salts, M199E; sterile filtered; 37°C) was applied to the luminal surface and incubated at room temperature for 10 minutes. The aorta was held in a 'boat' made from aluminium foil to ensure the collagenase solution bathed the luminal surface of the vessel and did not come into contact with the cut surfaces. Following incubation, the collagenase solution was gently aspirated and transferred to a sterile 15 ml tube for centrifugation (300 x g, 5 min, 20°C). Resuspended cells were cultured in M199E supplemented with 20% horse serum, 100 U/ml penicillin and 100 U/ml streptomycin.

Optimisation of culture conditions for EAoECs

The compositions of the media used in these studies are detailed in Table 1. Pooled isolates of EAoECs were split and cultured under identical conditions in either M199E or complete EGM2, both supplemented with 20% horse serum and antibiotics. Cells were observed under phase contrast light microscopy for growth characteristics and morphology. Growth rate was quantified by measuring proliferation as described in the main manuscript. Having observed that cells cultured in complete EGM2 had an elongated morphology, separate batches of 3 to 4 pooled isolates of EAoECs were split into tissue culture flasks (25 cm^2) and cultured in complete EGM2 or EBM. Morphology was quantified by measuring cell length and width from phase contrast images of confluent cells. Following the observation that culture in EBM led to morphology more consistent with quiescent endothelial cells, culture conditions were further optimised by taking pooled isolates of EAoECs and splitting as above to culture in EBM alone or EBM plus endothelial cell growth supplement (ECGS). Cells cultured in EBM and EBM plus ECGS were then assessed for their behaviour in the tube formation assay. Cells cultured in EBM were also assessed for their proliferation rate in response to different concentrations of equine serum.

Western blotting

EAoECs were plated onto gelatin-coated 6-well plates (300,000 cells/well) and grown to confluence. Cells were serum-deprived in EGM2 + 1% horse serum for 1 hour. The medium was aspirated, replaced with treatments (made up in EGM2 + 1% horse serum) and incubated for the relevant time (as indicated in the figure legends). Treatments were used at concentrations that have been fully optimised in our human EC studies. Cells were lysed in protein lysis buffer (76.5 mM Tris.HCl, 10% Glycerol, 2% SDS, pH 6.8 with 10 µl/ml (v/v) protease and phosphatase inhibitor cocktail + 25 U/ml Basemuncher; Expedeon). Protein contents of lysates were estimated using a bicinchoninic acid (BCA) protein assay detection kit (Pierce; ThermoScientific, UK). Equal amounts of protein were separated by sodium dodecyl sulfate polyacrylamide gel electrophoresis (SDS-PAGE; 10% Mini-PROTEAN TGX Precast Protein Gels) using a Protean® electrophoresis system (Bio-Rad) and resolved proteins transferred to polvinylidine fluoride (PVDF) membrane (0.45 µm) under semi-dry conditions (Bio-Rad) as described previously [1]. PVDF membranes were blocked for 1 hour at room temperature in Tris-buffered saline with Tween (TBST; 50 mM Tris, 150 mM NaCl, 0.02% (v/v) Tween 20, pH 7.4) containing 3% BSA.

For immunodetection membranes were probed with primary antibody overnight (4˚C; supplemental table 2) followed by HRP- or fluorescent-conjugated secondary antibody (1 hour; room temperature). Proteins were detected by enhanced chemiluminescence or directly using an automated imaging instrument (ChemiDoc™ MP, Bio-Rad).
Band densities were semi-quantified by densitometry using Image J. Values were normalised to loading controls and data are expressed as fold change compared to experimental control.

Name	M199E	EBM	EBM + ECGS	EBM + VEGF	Complete EGM2
Base medium	M199 with Earle's salts	Promocell Endothelial cell Basal Medium 2	Promocell Endothelial cell Basal Medium 2	Promocell Endothelial cell Basal Medium 2	Promocell Endothelial cell Basal Medium 2
Growth supplements	Endothelial Cell Growth Supplement (20 μg/ml)	None	Endothelial Cell Growth Supplement (20 μg/ml)	VEGF-A (0.5 ng/ml)	EGF (5 ng/ml)<br>FGF-2 (10 ng/ml)<br>IGF (20 ng/ml)<br>VEGF-A (0.5 ng/ml)<br>Ascorbic Acid (1 μg/ml)<br>Heparin (22.5 μg/ml)<br>Hydrocortisone (0.2 μg/ml)
Serum	20% horse serum	20% horse serum	20% horse serum	20% horse serum	20% horse serum
Antibiotics	100 U/ml penicillin<br>100 U/ml streptomycin	100 U/ml penicillin<br>100 U/ml streptomycin	100 U/ml penicillin<br>100 U/ml streptomycin	100 U/ml penicillin<br>100 U/ml streptomycin	100 U/ml penicillin<br>100 U/ml streptomycin

Table S1. Media compositions; VEGF-A (vascular endothelial growth factor), EGF (epithelial growth factor), FGF-2 (fibroblast growth factor-2), IGF (insulin-like growth factor).

Application	Primary/ secondary	Target	Clone	Host	Conjugate	Concentration	Diluent	Supplier
Western blotting	Primary	pERK 1/2	Polyclonal	Rabbit	None	1:1000	5% BSA in TBST	Cell Signalling Technologies
Western blotting	Primary	Total ERK	Polyclonal	Rabbit	None	1:1000	5% BSA in TBST	Santa Cruz Biotechnology
Western blotting	Primary	B-actin	ACTNO5	Mouse	None	1:1500	5% BSA in TBST	Abcam
Western blotting	Secondary	Rabbit	Polyclonal	Goat	HRP	1:5000	0.2% BSA in TBST	Cell Signalling Technologies
Western blotting	Secondary	Mouse	Polyclonal	Goat	Alexa Fluor 488	1:5000	0.2% BSA in TBST	Invitrogen
IF	Primary	VWF	Polyclonal	Rabbit	None	1:1000	PGAS	Dako
IF	Primary	VE-cadherin	55-7H1	Mouse	None	1:1000	PGAS	Invitrogen

IF	Primary	Smoothelin	Polyclonal	Rabbit	None	1:100	PGAS	Santa Cruz Biotechnology
IF	Secondary	Rabbit	Polyclonal	Goat	Alexa Fluor 488	1:500	PGAS	Invitrogen
IF	Secondary	Rabbit	Polyclonal	Goat	Alexa Fluor 568	1:500	PGAS	Invitrogen
IF	Secondary	Mouse	Polyclonal	Goat	Alexa Fluor 488	1:500	PGAS	Invitrogen
IF	Secondary	Mouse	Polyclonal	Goat	Alexa Fluor 568	1:500	PGAS	Invitrogen
IF	Secondary	Rat	Polyclonal	Goat	Alexa Fluor 488	1:500	PGAS	Invitrogen
En face	Primary	VWF	Polyclonal	Rabbit	None	1:1000	5% BSA in PBST	Dako
En face	Primary	VE-cadherin	55-7H1	Mouse	None	1:50	5% BSA in PBST	Invitrogen
En face	Secondary	Rabbit	Polyclonal	Goat	Alexa Fluor 488	1:500	PBST	Invitrogen
En face	Secondary	Mouse	Polyclonal	Goat	Alexa Fluor 568	1:500	PBST	Invitrogen
FACS	Primary	VE-cadherin	55-7H1	Mouse	PE	1:10	1% BSA in PBS	BD Biosciences
FACS	Primary	Isotype control	P3.6.2.8.1	Mouse	PE	1:25	1% BSA in PBS	eBioscience
Endothelial markers with no equine cross reactivity								
IF	Primary	Mouse CD31	MEC13.3	Rat	None	1:200	PGAS	BD Biosciences
En face	Primary	Rat CD31	TLD3-A12	Mouse	None	1:200	PGAS	Sigma-Aldrich
IF	Primary	Human CD31	89C2	Mouse	None	1:200	PGAS	Cell Signalling Technologies
IF	Primary	Human CD31	JC/70A	Mouse	None	1:25	PGAS	Novus Bio
IF	Primary	Human CD31	Polyclonal	Rabbit	None	1:100	PGAS	Abcam
IF	Primary	Human CD31	Polyclonal	Rabbit	None	1:200	PGAS	Biorbyt
IF	Primary	VE-cadherin	Polyclonal	Rabbit	None	1:500	PGAS	Unknown
IF	Primary	VEGFR2	55B11	Rabbit	None	1:500	PGAS	Cell Signalling Technologies
IF	Primary	Claudin-5	A-12	Mouse	None	1:60	PGAS	Santa Cruz Biotechnology
IF	Primary	ZO-1	R40.76	Rat	None	1:60	PGAS	Santa Cruz Biotechnology
IF	Primary	Ang-2	MM0020-IF29	Mouse	None	1:50	PGAS	Abcam

Table S2. Antibody details: BSA (bovine serum albumin), TBST (tris buffered saline with 0.02% Tween), IF (immunofluorescence), PGAS (phosphate, gelatin and saponin solution; 0.2 % gelatin, 0.02 % saponin, 0.02 % sodium azide in PBS), PBST (phosphate buffered saline with 0.1% triton-X).

cDNA synthesis mastermix (µl per 10 µl)		PCR mastermix (µl per 18 µl)	
RT buffer	2	Mastermix	10
dNTP Mix	0.8	Primers (F+R)	2
RT random primers	2	Nuclease-free water	6
Reverse transcriptase	1		
Nuclease-free water	4.2		

Table S3. Mastermix composition for cDNA synthesis and PCR; RT (reverse transcriptase), dNTP (deoxyribonucleotide triphosphate), F + R (forward and reverse).

qPCR cycling conditions			
Initial denaturation	94°C	2 min	1 x
Denaturation	94°C	15 sec	40 x
Annealing and extension	60°C	1 min	
Plate read			
Melt curve	60°C - 95°C; 0.5°C increment	5 sec	70 x
Plate read			

Table S4. PCR cycling conditions.

Gene	Sequence (forward, reverse)
Equine β-actin	ATGGATGATGATATCGCCG<br>CACGTATGAGTCCTTCTGG
Equine FGFR1	GATGATGCGGGATTGTTGGC<br>GAGCTTCGGGTGTCAGGAAA
Equine FGFR2	TCATCTGCCTGGTTGTGGTC<br>GATCATGGCGGCATCTCTCA
Equine VEGFR1	CGCTTCACCTGGACTGAGAG<br>GAGGAGAGCAGCACGAGATC
Equine VEGFR2	ATCTCTGGTCGTGAATGTC<br>CAATACCAGTGGATGTGATG
Equine NRP1	GACCGAAACTGGATGCCTGA<br>TCTTCCAGTCCGAACCGTTG
Human β-actin	GACAGGATGCAGAAGGAGATTACT

	TGATCCACATCTGCTGGAAGGT
Human FGFR1	CAAACCAAACCGTATGCCCG<br>AGGTGGCATAACGGACCTTG
Human FGFR2	CACGACCAAGAAGCCAGACT<br>GGTGTCTGCCGTTGAAGAGA
Human VEGFR1	CCATCAGCAGTTCCACCACT<br>CTTCTGGTTGGTGGCTTTGC
Human VEGFR2	GCAGGGGACAGAGGGACTTG<br>GAGGCCATCGCTGCACTCA
Human NRP1	GCCAGAGGAGTACGATCAGC<br>TCATCCACAGCAATCCCACC

Table S5. PCR primer sequences.

Supplementary figures

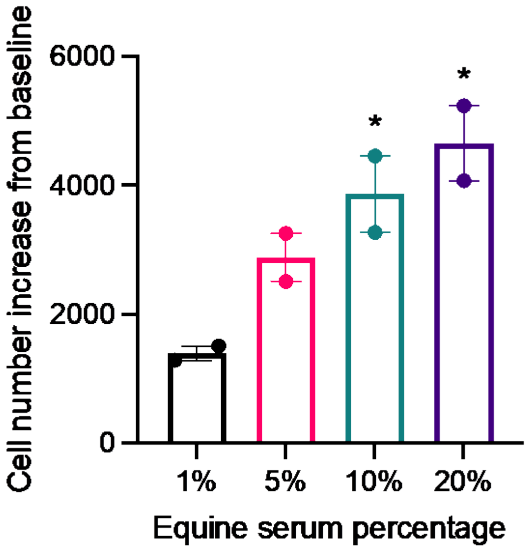

Supplementary Figure S1. Effects of equine serum on EAoEC proliferation rate over 48 hours (*p<0.05; One-way ANOVA with Dunnett's multiple comparisons test compared to 1%; mean ± S.E.M for n = 2 biological replicates with 3 technical repeats).

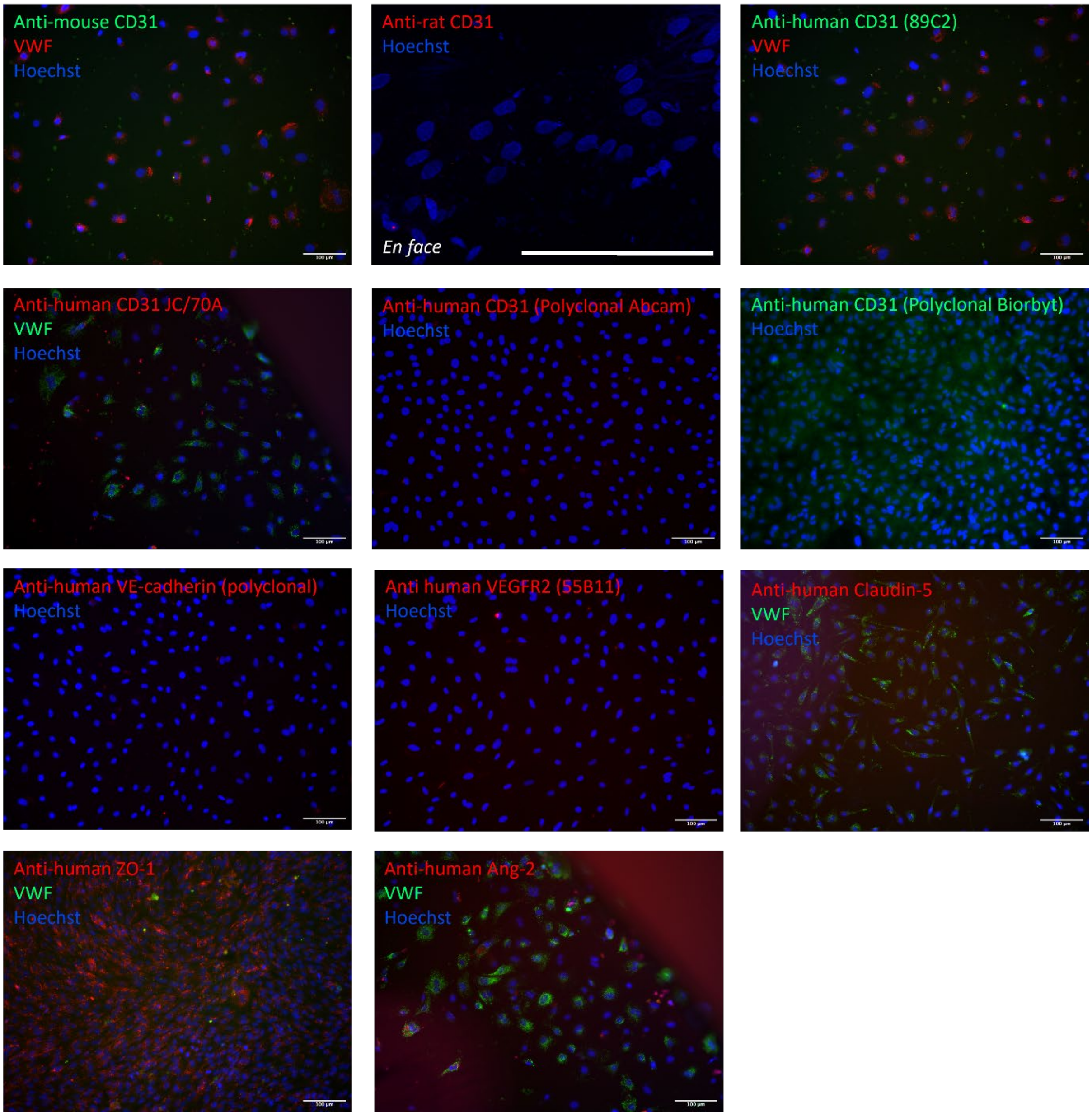

Supplementary Figure S2. Representative immunofluorescent images of cultured EAoECs or *en face* preparation of equine intercostal artery illustrating lack of cross reactivity, or non-specific staining, when using antibodies targeting specific endothelial cell markers. In some cases, cells were counterstained with VWF to confirm endothelial nature. Scale bars 100 μm.

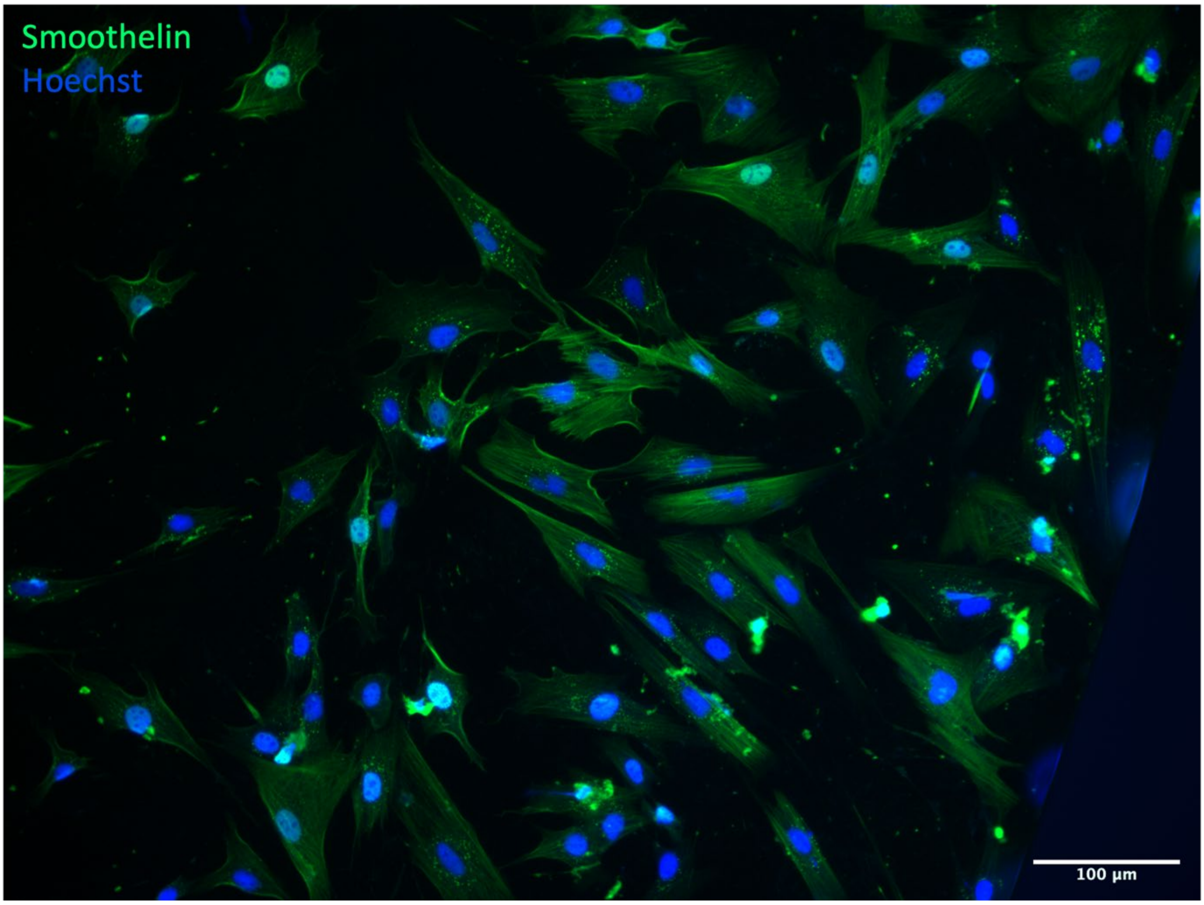

Supplementary Figure S3. Immunofluorescent image of a mixed population of cells (EC-poor) isolated from the equine aorta. Cells were fixed, permeabilised, blocked and stained using an antibody against smoothelin, a specific marker of vascular smooth muscle cells. Scale bar 100 μm.

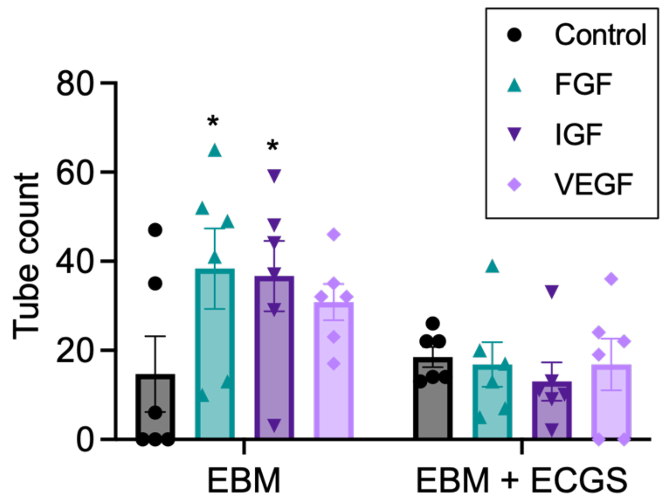

Supplementary Figure S4. EAoECs were exposed to FGF-2 (10 ng/ml), insulin-like growth factor (IGF; 50 ng/ml) or VEGF-A (25 ng/ml) for 16 hours and tube formation analysed. The pro-angiogenic response of ECs to growth factors was only observed with cells cultured in EBM and not those cultured in EBM supplemented with endothelial cell growth supplement (ECGS; *p = 0.03 (FGF-2) and *p = 0.05 (IGF); Two-way ANOVA with Dunnett's multiple comparisons test compared to control. Data are mean ± S.E.M for n = 6 technical replicates.

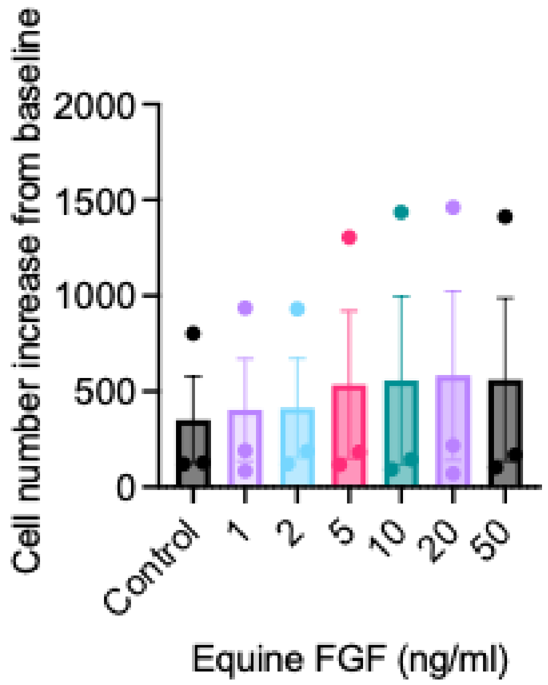

Supplementary Figure S5. EAoECs were exposed to FGF-2 at the indicated concentrations for 18 hours and cell proliferation analysed. No significant increase in cell number from control was identified at this time point. Data are mean ± S.E.M (n = 3 biological replicates) and were analysed by repeated measures one-way ANOVA with Dunnett's multiple comparisons test.

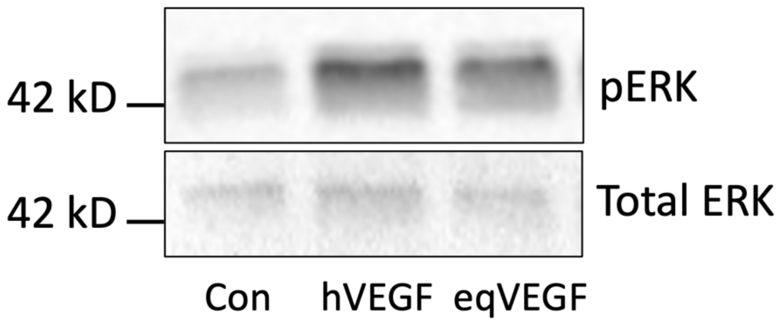

Supplementary Figure S6. Western blots to illustrate effectiveness of both human (h) and equine (eq) VEGF-A (25 ng/ml; 10 minutes) at activating ERK phosphorylation in human umbilical vein endothelial cells (HUVEC).

1. Faulkner, A., et al., *Context-dependent regulation of endothelial cell metabolism: differential effects of the PPARβ/δ agonist GW0742 and VEGF-A.* Scientific Reports, 2020. **10**(1): p. 7849.
